# Tetra-Repeat Microsatellite Markers for the Masu Salmon (*Oncorhynchus masou masou*) and Its Application in Cross-Subspecies Amplification

**DOI:** 10.3390/ijms141123153

**Published:** 2013-11-21

**Authors:** Shoichiro Yamamoto, Tadahide Kurokawa, Masashi Sekino, Motoshige Yasuike, Kenji Saitoh

**Affiliations:** 1 National Research Institute of Aquaculture, Fisheries Research Agency, Nikko, Tochigi 321-1661, Japan; 2 Tohoku National Fisheries Research Institute, Fisheries Research Agency, Shiogama, Miyagi 985-0001, Japan; E-Mail: kurokawa@affrc.go.jp; 3 National Research Institute of Fisheries Science, Fisheries Research Agency, Yokohama, Kanagawa 236-8648, Japan; E-Mails: sekino@affrc.go.jp (M.S.); yasuike@affrc.go.jp (M.Y.); ksaitoh@affrc.go.jp (K.S.)

**Keywords:** short tandem repeat, simple sequence repeat, 454 pyrosequencing, next-generation sequencer, *Oncorhynchus masou*

## Abstract

We developed tetranucleotide-repeat microsatellite markers for the masu salmon (*Oncorhynchus masou*) complex. 454 pyrosequencing was used to discover repeat motifs, and seven polymorphic microsatellite-primer sets were identified. The number of alleles detected at each locus ranged from four to 24 and the expected heterozygosity varied from 0.57 to 0.92. Cross-subspecies amplification for *O. m. masou*, *O. m. ishikawae* and *O. m.* subsp. was successful. These microsatellites can be utilized in studies of genetic structure, genetic diversity, and intra- and inter-subspecific hybridization, making a contribution to conservation and management of the *Oncorhynchus masou* complex.

## 1. Introduction

The masu salmon (*Oncorhynchus masou*) subspecies-complex (Salmonidae) is widely distributed in Far East Asia [1] and consists of four subspecies [2,3]. The subspecies are based on zoogeographic patterns and morphological characteristics; *O. m. masou* (Japanese name: sakura-masu), *O. m. ishikawae* (amago), *O. m.* subsp. (biwa-masu), and *O. m. formosanus. O. m. masou* and *O. m. ishikawae* have allopatric distributions, and are distinguishable by the spot-color pattern on their lateral sides: the former has black-spots while the latter has red-spots with sparse black-spots. *O. m.* subsp. is endemic to the Lake Biwa basin, central Honshu Island, Japan. Mitochondrial DNA analysis indicated that *O. m.* subsp. has a sister relationship with *O. m. masou* and *O. m. ishikawae* [4]. *O. m. formosanus*, which is endemic to Taiwan, has the world’s southernmost distribution among the native salmonid fishes.

The masu salmon subspecies-complex supports important recreational and commercial fisheries throughout the Japanese archipelago. Hatchery-reared masu salmon have been released into many rivers in Japan to enhance the fish stocks available to local fishermen and recreational anglers. However, their genetic integrities and diversities have been ignored, resulting in accelerated hybridizations between wild and hatchery fish within the subspecies [5,6] and among subspecies [7,8]. In addition, habitat fragmentations by artificial damming occur frequently in Japanese rivers, causing reduced population sizes and genetic diversities of salmonid populations [5,9,10]. *O. m.* subsp. is “near threatened (NT)” on the red list of the ministry of the environment, Japan (2007) [11]. *O. m. formosanus* is listed as an endangered species by the Taiwanese government, and as a critically endangered species by the International Union for Conservation of Nature (IUCN) [12–14].

Several dinucleotide-type microsatellite markers for masu salmon have been reported [15]. At present, however, we do not know whether these loci can be used across the masu salmon subspecies-complex. In addition, it will be useful to develop microsatellite markers with larger repeat motifs which allow easier genotyping than dinucleotide microsatellites. In this study, we performed 454 pyrosequencing [GS-FLX+ System, 454 Life Sciences by Roche Diagnostics (Branford, CT, USA)] to isolate novel microsatellite markers from the *O. m. masou* genome, and evaluated their potential for conservation genetic applications, such as cross-subspecies amplifications and population genetic analyses.

## 2. Results and Discussion

Seven polymorphic microsatellite loci were isolated and deposited in GenBank (AB851460-AB851466; Table 1). The number of alleles detected at each locus ranged from four (OMAS-10) to 24 (OMAS-4) and the expected heterozygosity varied from 0.57 (OMAS-10) to 0.92 (OMAS-5). The intrapopulation diversities differed, with the mean number of alleles per population varying between 4.43 (Miya River: *O. m. ishikawae*) and 11.29 (Chitose River: *O. m. masou*), and the expected heterozygosity varied between 0.60 (Miya River) and 0.80 (Chitose River; Table 2). Among 27 Hardy-Weinberg equilibrium (HWE) tests, where OMAS-10 marker in *O. m. ishikawae* was omitted due to no allelic variation, six were significant after the false discovery rate correction of significant level for multiple comparisons. The observed departures, however, were neither locus nor population specific; therefore, we could not determine the cause of the HWE deviations. No marker pair showed evidence of departure from linkage equilibrium, indicating no significant allelic association between the markers. These microsatellites represent useful tools for population genetic applications, such as stock identification in the *Oncorhynchus masou* complex.

## 3. Experimental Section

### 3.1. Isolation of Microsatellite Markers

Total genomic DNA from *O. m. masou* (blood cell) was extracted by proteinase K digestion, phenol/chloroform extraction, and ethanol precipitation. We prepared a shotgun library from 1.0 μg of genomic DNA following the 454-Roche protocol. Pyrosequencing was conducted on one-fourth picotiter plate. We searched for microsatellites and microsatellite-flanking PCR primer sequences using Auto-Primer [16]. A 454 run generated 175,689 sequence reads (63,019,585 bp) from *ca.* 5.0 × 10^5^ beads with clonally amplified DNA, resulting in 1937 contigs and 129,759 singletons. Auto-Primer proposed 242 PCR primer sets for tetra-nucleotide microsatellites. We selected 18 primer sets designed for amplification of “perfect-type” tetra-nucleotide repeats (i.e., repeat arrays without interruption by a non-repeat element); 11 were rejected because of poor amplification or difficulty in allele scoring. The remaining seven sets were applicable for estimation of polymorphism (Table 1).

### 3.2. PCR Amplification and Genotyping

Three wild population samples (*O. m. masou* from the Chitose River, Hokkaido Island, Japan; *O. m. ishikawae* from the Miya River, central Honshu, Japan; and *O. m.* subsp. from Lake Biwa, central Honshu, Japan) and one hatchery-reared stock sample (*O. m. masou* from the Ohara River, northern Honshu, Japan) were used for polymorphism detection. After conjugating 6-FAM, HEX, NED or PET fluorescent dyes (Applied Biosystems, Life Technologies, Carlsbad, CA, USA) to the 5′-end of each forward primer, we performed multiplex PCR for the seven selected primer sets using a Qiagen multiplex PCR kit (Qiagen, Limburg, The Netherlands). The 10-μL reaction mixture contained 1× Qiagen multiplex PCR master mix, 0.2 μM of each primer, 2 μL of distilled water, and 1 μL of DNA solution. Amplifications were carried out in a GeneAmp PCR System 2700 thermal cycler (Applied Biosystems), according to the supplier’s instructions (Qiagen multiplex PCR kit; Qiagen): initial denaturation at 95 °C for 15 min; followed by 30 cycles of denaturation at 94 °C (30 s), annealing (60 °C, 90 s) and extension (72 °C, 60 s); with a final extension (60 °C, 30 min). PCR fragments and GeneScan 600LIZ size standards were resolved on an ABI PRISM 3730*xl* sequencer (Applied Biosystems, Life Technologies). Allele size was determined using GeneMapper v4.1 (Applied Biosystems, Life Technologies).

### 3.3. Data Analysis

Polymorphism statistics, including the observed/expected heterozygosities, were calculated in Arlequin v3.5 [17]. The Hardy-Weinberg equilibrium of markers/populations was tested using Fisher’s exact test with the Markov chain method (Markov chain steps: 10^5^; Dememorization: 10^5^). We also examined linkage equilibrium between markers (likelihood-ratio test: 10^4^ permutations). Critical significance levels for multiple testing were adjusted following the false discovery rate [18].

## 4. Conclusions

We report the isolation and characterization of seven polymorphic microsatellite loci for the *Oncorhynchus masou* complex. All seven markers used were polymorphic. Cross-subspecies amplification for *O. m. masou*, *O. m. ishikawae* and *O. m.* subsp. was successful. These microsatellites can be utilized in studies of genetic structure, genetic diversity, and intra- and inter-subspecific hybridization, making a contribution to conservation and management of the *Oncorhynchus masou* complex.

## Figures and Tables

**Table 1:** Tetra-nucleotide microsatellite markers for *Oncorhynchus masou* complex.

Marker	Motif *1		Primer sequences (5′-3′) *2	*T*a *3	*R*_A_ (bp) *4	GenBank No.
OMAS-3	(AGAC)_14_	F-N	AGAGACAGATAGAGCCAGCCAG	60	127–199	AB851460
		R	TGATGAACGTTACGATTGGAAG			
OMAS-4	(CACT)_12_	F-P	TGCACATAAATTGAAGGCAAAC	60	137–249	AB851461
		R	GAGTCACCTGTCCCTCAGTACC			
OMAS-5	(TCTG)_13_	F-F	TTTTGCTGGTGACTCCCTAAAT	60	236–348	AB851462
		R	ATTTGCAGAGGGAAACAGACAT			
OMAS-7	(AGAC)_16_	F-N	GGGAAAGAAAGGAGATTGAGAGA	60	235–295	AB851463
		R	GACCCTGGTAGAACTGCAAACT			
OMAS-10	(GAGG)_6_	F-V	AGCAAAGGGAGATAAGGTAGGG	60	305–313	AB851464
		R	CATCTTCATTCAGAGGGGTAGG			
OMAS-14	(GAGT)_7_	F-F	ATTGTTAGAGCGGGAGACGATA	60	98–138	AB851465
		R	TCCCCAGAATTGTTAGCTGAGT			
OMAS-18	(ATCA)_9_	F-V	CACACAAAGAAAACCTTGAATGA	60	113–157	AB851466
		R	AACGTGACTGCACAACAAAGTT			

*1 Repeat counts are based on the individual from which the microsatellite-primer sequences were obtained;

*2 The forward primer of each primer set was 5′-end-labeled with 6-FAM (denoted as F-F), VIC (F-V), NED (F-N) or PET (F-P) fluorescent dye;

*3 Optimized annealing temperature.

*4 Range of allele sizes.

**Table 2:** Genetic diversity statistics for the three subspecies of *Oncorhynchus masou*.

Subspecies	River/Lake	Statistics of polymorphisms *1	OMAS-3	OMAS-4	OMAS-5	OMAS-7	OMAS-10	OMAS-14	OMAS-18
*Oncorhynchus masou masou*	Chitose River	*H*_o_	0.86	0.93	0.73	0.90	0.50	0.67	0.31
		*H*_E_	0.89	0.90	0.84	0.86	0.62	0.75	0.74
		HW_*P*	0.12	0.01 ^*^	0.00 ^*^	0.09	0.10	0.02	0.00 ^*^
		Number of Alleles	15	17	17	12	3	8	7
		*n*	43	42	40	42	42	43	35

*Oncorhynchus masou ishikawae*	Miya River	*H*_o_	0.46	0.83	0.75	0.24	-	0.60	0.15
		*H*_E_	0.44	0.85	0.75	0.61	-	0.44	0.14
		HW_*P*	1.00	0.00 ^*^	0.02	0.00 ^*^	-	0.02	1.00
		Number of Alleles	5	8	6	6	1	2	3
		*n*	48	48	48	41	48	48	48

*Oncorhynchus masou* subsp.	Lake Biwa	*H*_o_	0.78	0.87	0.78	0.78	0.06	0.53	0.81
		*H*_E_	0.81	0.89	0.86	0.85	0.06	0.62	0.78
		HW_*P*	0.66	0.80	0.10	0.11	1.00	0.60	0.98
		Number of Alleles	9	14	11	9	2	7	7
		*n*	32	31	32	32	32	32	32

*Oncorhynchus masou masou* (hatchery stock)	Oohara River	*H*_o_	0.79	0.64	0.84	0.71	0.56	0.79	0.14
		*H*_E_	0.71	0.69	0.86	0.67	0.53	0.68	0.27
		HW_*P*	0.56	0.47	0.02	0.69	0.91	0.37	0.00 ^*^
		Number of Alleles	7	6	8	4	3	4	3
		*n*	39	39	38	38	39	39	37

Average	-	*H*_o_	0.72	0.82	0.77	0.66	0.37	0.65	0.35
		*H*_E_	0.71	0.83	0.83	0.75	0.40	0.62	0.48
		Number of Alleles	9	11	11	8	2	5	5

*1 *H*o: observed heterozygosity; *H*_E_: expected heterozygosity; HW_*P*: probability value of Hardy-Weinberg equilibrium (HWE) testing. Significant HWE departure after the false discovery rate correction of significance level for multiple comparisons is indicated by an asterisk; *n*: number of genotyped individuals.
